# Identifying nurse staffing research in Medline: development and testing of empirically derived search strategies with the PubMed interface

**DOI:** 10.1186/1471-2288-10-76

**Published:** 2010-08-23

**Authors:** Michael Simon, Elke Hausner, Susan F Klaus, Nancy E Dunton

**Affiliations:** 1 School of Nursing, University of Kansas Medical Center, 3901 Rainbow Blvd, Kansas City, Kansas 66160, USA; 2 Institute for Quality and Efficiency in Health Care (IQWiG), Dillenburger Str. 27, 51105 Köln

## Abstract

**Background:**

The identification of health services research in databases such as PubMed/Medline is a cumbersome task. This task becomes even more difficult if the field of interest involves the use of diverse methods and data sources, as is the case with nurse staffing research. This type of research investigates the association between nurse staffing parameters and nursing and patient outcomes. A comprehensively developed search strategy may help identify nurse staffing research in PubMed/Medline.

**Methods:**

A set of relevant references in PubMed/Medline was identified by means of three systematic reviews. This development set was used to detect candidate free-text and MeSH terms. The frequency of these terms was compared to a random sample from PubMed/Medline in order to identify terms specific to nurse staffing research, which were then used to develop a sensitive, precise and balanced search strategy. To determine their precision, the newly developed search strategies were tested against a) the pool of relevant references extracted from the systematic reviews, b) a reference set identified from an electronic journal screening, and c) a sample from PubMed/Medline. Finally, all newly developed strategies were compared to PubMed's Health Services Research Queries (PubMed's HSR Queries).

**Results:**

The sensitivities of the newly developed search strategies were almost 100% in all of the three test sets applied; precision ranged from 6.1% to 32.0%. PubMed's HSR queries were less sensitive (83.3% to 88.2%) than the new search strategies. Only minor differences in precision were found (5.0% to 32.0%).

**Conclusions:**

As with other literature on health services research, nurse staffing studies are difficult to identify in PubMed/Medline. Depending on the purpose of the search, researchers can choose between high sensitivity and retrieval of a large number of references or high precision, i.e. and an increased risk of missing relevant references, respectively. More standardized terminology (e.g. by consistent use of the term "nurse staffing") could improve the precision of future searches in this field. Empirically selected search terms can help to develop effective search strategies. The high consistency between all test sets confirmed the validity of our approach.

## Background

PubMed/Medline contains more than 18 million references. The identification of relevant literature in this wide-ranging source is of great importance to researchers in remaining up-to-date with the latest developments in the field of interest, as well as in conducting comprehensive literature reviews. *"Search filters are collections of search terms intended to capture frequently sought research methods, such as randomized controlled trials, or aspects of health care" *[1]. While this definition includes methods filters for certain common research methods such as randomized controlled trials (RCTs) [2-5] and systematic reviews [3,6-9], the identification of relevant literature in fields with less standardized methods such as health services research remains a cumbersome task. Furthermore, methods filters need to be complemented with terms of the topic of interest to identify the relevant literature. The development of the topic-specific part of the search strategy usually consists of an arbitrary selection of terms. Few studies have been conducted with the aim of systematically identifying this topic-specific part [10-12]. An approach guiding the selection of relevant terms could help researchers develop search strategies in a more objective and systematic manner for both topic and methods-related searches.

Nurse staffing research investigates the association between nurse staffing parameters and nursing and patient outcomes [13]. The basic question in nurse staffing research is which nurse-to-patient ratios result in high-quality patient care. Although previous research in this field has largely been observational in nature, a wide range of statistical methods and data sources are used [14], which makes it difficult to identify the relevant literature effectively. Empirically tested search strategies support the identification of literature in an effective and efficient manner [1,15], and are used in searches conducted in the production of systematic reviews and in the creation of automatic e-mail updates with PubMed's My NCBI.

To date, the development of empirically tested search strategies has been focused on identifying certain study types, such as RCTs and systematic reviews. Most research on search filters has tested the developed search strategy against a defined set of references from a hand search (gold standard) or other systematic reviews (quasi-gold standard) [1,15-24]. An approach based on a set of relevant references identifying appropriate terms and then testing the developed strategy against several test sets could be used for search strategy development in general, beyond its sole use in the development of methods filters.

In the context of systematic reviews, the number of relevant references on a given topic in a database is a matter of particular interest. An estimate of the number of relevant references in the database could be used for resource planning purposes within the framework of comprehensive systematic reviews.

The four aims of this study were to:

1. develop search strategies to identify primary publications on nurse staffing research in PubMed/Medline

2. test the search strategies against a set of relevant references from different sources

3. compare the search strategies with PubMed's health services research queries (PubMed HSR Queries)

4. estimate the number of relevant nurse staffing references in PubMed/Medline

## Methods

### Search strategy development

Three search strategies were developed, targeting either the highest sensitivity ('sensitive strategy'), the highest precision ('precise strategy'), or a balance between sensitivity and precision ('balanced strategy'). In the context of search strategy development, sensitivity (or recall) is the number of relevant references retrieved divided by the number of all relevant references. A search with a sensitivity of 1, for instance retrieves all relevant references, while a search with a sensitivity of 0.5 retrieves half of all relevant references. Precision is the fraction of the relevant references of all retrieved references. For example, a precision of 0.33 means that a third of all retrieved references are relevant to the topic of interest. Search strategies solely aimed at sensitivity or precision target the extremes of the inverse relationship of these two parameters. A balanced strategy attempts to achieve both aims: to achieve high sensitivity without losing too much precision and vice versa. Balancing is based on the iterative addition and removal of parts of the search strategy to determine a balance between sensitivity and specificity. However, this balance is not precisely defined and remains a vague concept.

The employed development process of the search strategy includes four sets of references to define and test the developed strategies. Two sets of references were used for the development of the search strategies, a development and a population set.

The *development set *was used to identify and evaluate the sensitivity of free-text terms (title, abstract) and Medical Subject Heading (MeSH) terms. The development set consisted of a pool of 78 relevant papers from PubMed/Medline, identified in three relevant systematic reviews investigating the association between nurse staffing and patient outcomes [13,25,26]. Systematic reviews have previously been used to identify relevant references for search filter development [24]. Well-conducted systematic reviews employ comprehensive searches in various databases and are often complemented by hand searches. A set of references created by merging relevant references from different systematic reviews can be assumed to represent the total population of relevant references. The selection of systematic reviews was not based on a systematic search but on a priori knowledge of the field. The systematic reviews were selected because, to our knowledge, they employed the most comprehensive searches so far targeting nurse staffing research [13,25]. Only those studies critically appraised and included in the systematic reviews and available in PubMed/Medline were incorporated in the development set.

A *population set *consisting of a random sample of PubMed/Medline references was used to compare the frequency of terms with the highest sensitivity from the development set with the frequency in the overall PubMed/Medline population. For the sampling procedure we limited a PubMed/Medline search (using an empty search field) to the last 12 months (12/2007 to 12/2008) and saved the retrieval results as a PMID list. From this list a random sample of 10,000 references was drawn. References of the population set were not screened for relevance and all references were assumed to be not relevant.

A text-mining approach was used to identify potentially relevant free-text terms from the development set. The analysis was computed with the tm package [27] in R [28], which is a statistical computing language and graphics environment.

The tm package creates a term-document matrix consisting of all terms used in a set of references (78 in this case) and expresses the frequency of each term in each reference. The PubMed/Medline references in the development set contained 1,779 terms. Terms present in at least five percent of the references (359 candidate terms) of the development set were selected for additional exploration. To further decrease the number of candidate terms, the 25 most overrepresented terms from the development set compared to the population set were used to develop the free-text part of the search strategies in PubMed/Medline. "Overrepresented" was defined as the most widely used terms in the 78 references of the development set that were prevalent in 2% or fewer references of the population set. Table 1 shows the prevalence of these 25 terms in both data sets.

**Table 1:** Prevalence of terms in the development and population set

		development set n = 78		population set n = 10,000
	
	n	prevalence	n	prevalence
nurse	55	0.71	79	0.01
hospitals	50	0.64	114	0.01
staffing	49	0.63	10	0.00
nursing	45	0.58	186	0.02
nurses	39	0.50	114	0.01
staff	22	0.28	88	0.01
stay	21	0.27	146	0.01
registered	20	0.26	40	0.00
units	20	0.26	173	0.02
mix	17	0.22	17	0.00
relationships	17	0.22	181	0.02
organizational	15	0.19	30	0.00
ratios	16	0.21	173	0.02
odds	16	0.21	189	0.02
intensive	15	0.19	143	0.01
adjusted	14	0.18	175	0.02
teaching	13	0.17	52	0.01
falls	12	0.15	33	0.00
rns	10	0.13	8	0.00
satisfaction	11	0.14	143	0.01
skill	10	0.13	22	0.00
proportion	11	0.14	179	0.02
medicare	10	0.13	57	0.01
multivariate	11	0.14	189	0.02
tract	11	0.14	192	0.02

The text-mining approach applied worked reliably only for single word terms. MeSH terms often consist of multiple words including special characters, which lead to unexpected results. Due to this technical constraint, a simplified approach was applied to identify the 20 most frequent MeSH terms to be used in the search strategy. Terms were selected on the basis of their frequency in the development set and their relevance to the question.

The final stage of the search strategy development consisted of iterative queries in PubMed/Medline comparing different combinations of free-text and MeSH terms in order to develop the three search strategies: 1) sensitive, 2) precise, and 3) a balance between sensitivity and precision. The identification of the most effective combination of terms for the most sensitive, precise or balanced strategy is still effected manually. However, on the basis of the pre-selection of relevant and specific terms, this process is considerably shorter than a non-empirically informed development process. Table 2 shows the developed search strategies in PubMed/Medline. We provide a single-line syntax for PubMed (Table S1) and a syntax for OVID^SP ^Medline (untested, Table S2) as additional file 1.

**Table 2:** Empirically derived search strategies to identify nurse staffing research in PubMed

Sensitive	
1	staff[tiab] OR staffing[tiab] OR organizational[tiab] OR skill mix[tiab] OR length of stay[tiab] OR medicare[tiab]
2	"Nursing Staff, Hospital"[mh]
3	"Personnel Staffing and Scheduling"[mh]
4	"Intensive Care Units/manpower"[mh]
5	"Nursing Administration Research"[mh]
6	#1 OR #2 OR #3 OR #4 OR #5
7	"health services administration"[mh]
8	nurse[tiab] OR nurses[tiab] OR hospitals[tiab] OR nursing[tiab]
9	"hospital units"[mh]
10	#8 OR #9
11	#6 AND #7 AND #10
**Precise**	

1	"Outcome and Process Assessment (Health Care)" [mh]
2	"Hospital Units" [mh]
3	hospitals[tiab]
4	#1 OR #2 OR #3
5	nurse[tiab] or nurses[tiab]
6	staffing[tiab]
7	"Nursing Staff, Hospital" [mh]
8	#6 OR #7
9	outcomes[tiab]
10	#4 AND #5 AND #8 AND #9
**Balanced**	

1	"Outcome and Process Assessment (Health Care)" [mh]
2	"Hospital Units" [mh]
3	hospitals[tiab]
4	#1 OR #2 OR #3
5	(nurse[tiab] OR nurses[tiab]) AND staffing[tiab]
6	"Nursing Staff, Hospital" [mh]
7	#5 OR #6
8	#4 AND #7

### Testing the search strategies

The newly developed search strategies (Table 2) were tested against three reference sets: the development, precision, and journal screening sets. All tests were conducted with the PubMed interface. PubMed/Medline was chosen for its free accessibility. The search development and testing were conducted in December 2008.

The following formulas were used for the calculation of sensitivity (1), precision (2), and the number needed to read (3):

(1) $$Sensitivity=\frac{relevant\;records\;retrieved}{all\;relevant\;records}$$

(2) $$Precision=\frac{relevant\;records\;retrieved}{all\;retrieved\;records}$$

(3) $$Number\;needed\;to\;read(NNR)=\frac{all\;retrieved\;records}{relevant\;records\;retrieved}$$

The *development set *consisted of 78 relevant references from the three reviews. As we did not screen the retrieved references in PubMed/Medline for relevance, we calculated precision based on the conservative assumption that all references retrieved additionally were not relevant. Retrieval of results (recall) was limited to the time frame of the searches of the systematic review (1982 to 2006). This rough approximation results in a downward bias of precision and NNR in the development set, as relevant references in the non-screened references were ignored. If relevant references had been identified, precision and NNR would have been improved.

All search strategies tested (Sensitive, Precise, Balanced, PubMed HSR Sensitive, PubMed HSR Precise) were connected with the OR operator and limited to the time frame between 1982 and 2006. A random sample of 2,195 references was drawn from the retrieved 35,708 records and screened for relevance. This set was used to determine a less biased estimate of the precision of the search strategies and to estimate the overall number of relevant references. This estimation was based on the assumption that a joint search including all search strategies (with sensitivity of up to 1.00) should be able to capture all relevant studies in PubMed/Medline for the given time frame. Following this assumption it is possible to calculate an estimate for the number of relevant references in PubMed/Medline using the precision set. The relevant references of the precision set overlapped with the development set, except for one reference. This overlap can be expected for two reasons: 1) both sets target the same time frame, and 2) the development set was based on three comprehensive searches, which potentially captured all relevant records in this time frame.

The *journal screening set *was based on the assessment of all available abstracts of three journals relevant to nurse staffing research (Medical Care, Health Services Research, and Journal of Nursing Administration; issues 2006 to 2008; 1,274 references). The selection of journals was based on the frequency of relevant articles in each journal in the development set. The time frame of the latest search in the systematic reviews and the journal screening overlapped by six months, which resulted in one paper being included in both sets and two papers not being identified by the systematic reviews; we assume this was caused by the delay in full indexing in PubMed/Medline. There was no overlap of references between the journal screening and precision set.

The references retrieved from the precision and journal screening set were independently assessed for relevance by two of the authors (MS, SK). Eligibility criteria were based on the three systematic reviews (Table 3). Inconsistencies in the classification of references as relevant or non-relevant were resolved by consensus.

**Table 3:** Inclusion and exclusion criteria for the precision and journal screening set

Inclusion criteria:	• Studies investigating the association between staffing (e.g. nurse-to-patient ratio or work hours per patient or patient day) and a) nursing outcomes (e.g. job satisfaction, nurse vacancy rate, nurse turnover rate, nurse retention rate) or b) patient outcomes (e.g. mortality, adverse drug events, nurse quality outcomes, length of stay; patient satisfaction with nursing care)
Exclusion criteria:	• Studies not published in English
	• Studies including a target population of outpatients and patients in long-term care facilities
	• Studies with no information relevant to nurse staffing policies and strategies
	• Studies examining the contributions of advance practice nurses (nurse practitioners, nurse clinicians, certified nurse midwives, nurse anesthetists)
	• Administrative reports and single-hospital studies that did not include control comparisons and did not test an associative hypothesis
	• Systematic or non-systematic reviews
	• Editorials, letters, non-original research

**Figure 1:**
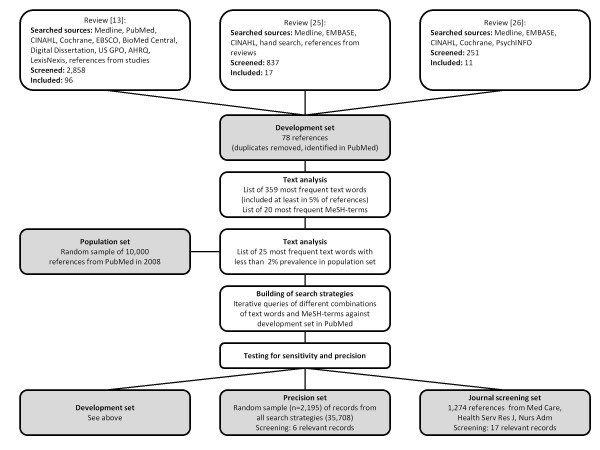
**Development process and testing of search strategies**.

The performance of the newly developed search strategies was compared to PubMed's sensitive and precise special queries for outcomes in health services research (PubMed HSR Queries) [29]. PubMed's HSR Queries are methods filters targeting health services research, including nurse staffing research. These were combined with the topic-specific terms from the newly developed strategies. Figure 1 outlines the development process and the testing of the search strategies.

## Results

### Performance of the newly developed strategies

The sensitive search strategy captured almost 100% of the relevant references in all test sets (Table 4), while the precise strategy captured between 6.1% and 32.0%. To identify a relevant paper from the retrieved references of the sensitive strategy, users would need to screen 297 references (NNR), while the precise strategy detected one relevant reference in every three.

**Table 4:** Sensitivity and precision of the search strategies tested

	Retrieved documents [relevant references]	Sensitivity (%)	Precision (%)	NNR*
*Development set (78 relevant references)*				

Sensitive strategy	28,893 [77]	98.7	0.2	483
Precise strategy	461 [40]	51.3	6.1	16
Balanced strategy	4,351 [62]	79.5	1.0	96
PubMed HSR Query sensitive	16,636 [68]	87.2	0.3	328
PubMed HSR Query precise	310 [20]	25.6	5.0	20

*Precision set (6 relevant references out of a total of 2,195)*				

Sensitive strategy	1,775 [6]	100	0.3	297
Precise strategy	34 [5]	83.3	14.7	7
Balanced strategy	278 [5]	83.3	1.8	56
PubMed HSR Query sensitive	1,017 [5]	83.3	0.5	203
PubMed HSR Query precise	20 [3]	50.0	15.0	7

*Journal screening set (17 relevant references out of a total of 1,274)*				

Sensitive strategy	210 [17]	100	8.1	12
Precise strategy	25 [8]	47.1	32.0	3
Balanced strategy	65 [13]	76.5	20.0	5
PubMed HSR Query sensitive	127 [15]	88.2	11.9	8
PubMed HSR Query precise	7 [3]	17.6	30.0	3

### Comparison with PubMed's HSR Queries

The newly developed search strategies had a higher sensitivity than PubMed's HSR Queries in all test sets (Table 4). In terms of precision, the two strategies performed within the same range in all of the three test sets.

### Overall estimate of relevant references on nurse staffing in PubMed/Medline

The precision set contained a total of 2,195 references, of which 6 were relevant references according to the eligibility criteria defined. On the basis of the precision set, an overall number of 97.6 [19.5-175.2] relevant references would be expected on nurse staffing for the time frame between 1982 and 2006.

## Discussion

The search strategies developed performed well in terms of sensitivity, with the expected pay-off for precision and vice versa. Depending on the objective of the search, all three strategies are suitable for specific purposes such as the use of sensitive strategies in systematic reviews or e-mail alerts.

All strategies were assessed against three different test sets. For the measurement of sensitivity, the development and journal screening sets produced similar test results, while the results of the precision set showed greater differences. We assume these differences were caused by the insufficient sample size of the precision set. Although 2,195 references do not appear to be a small sample size, for a given prevalence of 0.0027% of relevant references in the PubMed/Medline population, the sample is still small. Precision ranged from 0.2% to 6.1% in the development set, 0.3% to 14.7% in the precision set, and 8.1% to 32.0% in the journal screening set. Although the ranges varied considerably between the test sets, the overall pattern in the comparison of the search strategies remained consistent: the precise strategies performed better than the balanced strategies and sensitive strategies. Conceptually, the precision set was the closest to the true population. Even with 2,195 screened references, this approach lacked the accuracy to differentiate between strategies in terms of sensitivity. However, precision derived from this set was not hampered by the small sample size and produced less biased estimates for the PubMed/Medline population than the development and journal screening set.

The comparison of PubMed's HSR Queries with the newly developed strategies shows advantages for the latter strategies. This favourable assessment could be expected, due to the broader scope of the HSR Queries. Therefore it might be more important to consider performance comparison as a validation method for the developed strategies and the test sets used.

One of the strengths of the population set is the possibility to infer to the overall PubMed/Medline population and calculate the expected number of relevant references on the topic of interest. However, it should be taken into account that this estimate is based on the Medline references of PubMed/Medline and therefore ignores a small percentage of non-Medline references. Although limited by wide confidence intervals, when exclusively compared to the development and journal screening set, the estimate allows an inference of the overall number of relevant papers.

In addition to the aims outlined, the study employed a development process for search strategies, with some features that might be useful to search strategy development in general. While the identification of candidate terms and the testing of the strategy against the development set have previously been done in research on methods filters, in our opinion the population and precision sets employed are unique features of this study. These sets allow (1) search strategy developers to select terms that are not only frequently used in relevant publications but also specific to the topic of interest, and (2) to achieve more realistic precision estimates for the PubMed/Medline population. For search strategy developers, the frequency of terms should not be the sole criterion for the selection of a term for a search strategy. For example, the term "patients" is present in 65% of the references in the development set, but also in 77% in the population set, indicating a lack of specificity for the topic of interest. The population set enables the developer to preselect these specific terms in order to develop sensitive and precise searches.

Although the development process described could support the development of performance-oriented search strategies, in general some limitations apply to this study and the generalizability of the process.

We assumed that the selected systematic reviews used for building the development set are the most comprehensive reviews in the topic area. However, we cannot rule out that other reviews containing additional relevant references exist.

The search filters developed require references to be fully indexed in Medline and might not be able to fully capture citations in-process; this applies to many search filters [30] and also limits the use of search strategies as e-mail-update filters.

An untested search strategy without MeSH terms is provided in Additional file 1 (Table S3).

## Conclusions

As with other literature on health services research, nurse staffing studies are difficult to identify in PubMed/Medline. Even though sensitive search strategies result in a high level of sensitivity, the considerable number of non-relevant references is a burden. Depending on the purpose of the search, researchers can choose between high sensitivity or high precision, i.e. retrieval of a large number of references or an increased risk of missing relevant references, respectively. More standardized terminology (e.g. by consistent use of the term "nurse staffing") could improve the precision of future searches in this field.

The described development process for an empirical search strategy is a useful - though technically demanding - approach to building performance-oriented strategies. The similar sensitivities of the tested strategies in the development and journal screening set confirm the validity of this approach. The precision set can be used to provide more realistic precision estimates and to calculate the expected number of relevant references in the population set.

## Competing interests

The authors declare that they have no competing interests.

## Authors' contributions

MS coordinated the planning of the study, data collection, search strategy development, data analysis, results interpretation and drafting and revision of the manuscript. EH participated in the review of the literature, results interpretation, and revision of the manuscript. SK participated in data collection, results interpretation, and revision of the manuscript. ND participated in results interpretation and revision of the manuscript. All authors read and approved the final manuscript.

## Pre-publication history

The pre-publication history for this paper can be accessed here:

http://www.biomedcentral.com/1471-2288/10/76/prepub

## Supplementary Material

Additional file 1 **Single-line syntax for PubMed (Table A1) and untested syntax for OVIDSP for Medline (Table A2)**. The single line syntax of the search strategies for PubMed and the OVIDSP syntax (untested) and provided as a convenience to the reader. Click here for file
