# Supplementary material for: Identifying nurse staffing research in Medline: development and testing of empirically derived search strategies with the PubMed interface
Source: BMC Med Res Methodol. 2010 Aug 23;10:76. doi: 10.1186/1471-2288-10-76 (PMC2936389; doi:10.1186/1471-2288-10-76)
Supplement: Additional file 1 — Single-line syntax for PubMed (Table A1) and untested syntax for OVIDSP for Medline (Table A2). The single line syntax of the search strategies for PubMed and the OVIDSP syntax (untested) and provided as a convenience to the reader. [file 1471-2288-10-76-S1.DOC]

Table S1: Single line PubMed search filters

| Sensitive | ((staff[tiab] OR staffing[tiab] OR organizational[tiab] OR skill mix[tiab] OR length of stay[tiab] OR medicare[tiab]) OR "Nursing Staff, Hospital"[mh] OR "Personnel Staffing and Scheduling"[mh] OR "Intensive Care Units/manpower"[mh] OR "Nursing Administration Research"[mh]) AND ("health services administration"[MeSH Terms] AND (nurse[tiab] OR nurses[tiab] OR hospitals[tiab] OR nursing[tiab] OR "hospital units"[MeSH Terms])) |
| --- | --- |
| Precise | (("Outcome and Process Assessment (Health Care)"[mh] OR Hospital Units[mh] OR hospitals[tiab]) AND (((nurse[tiab] OR nurses[tiab]) AND staffing[tiab]) OR (nursing staff, hospital[mh]))) AND (outcomes[tiab]) |
| Balanced | ("Outcome and Process Assessment (Health Care)"[mh] OR Hospital Units[mh] OR hospitals[tiab]) AND (((nurse[tiab] OR nurses[tiab]) AND staffing[tiab]) OR (nursing staff, hospital[mh])) |

Table S2: Search filters for Ovid Medline (untested)

| Sensitive | 1 | (staff or staffing or organizational or skill mix or length of stay or medicare).ti,ab. |
| --- | --- | --- |
|  | 2 | Nursing Staff, Hospital/ |
|  | 3 | "Personnel Staffing and Scheduling"/ |
|  | 4 | exp Intensive Care Units/ma |
|  | 5 | Nursing Administration Research/ |
|  | 6 | or/1-5 |
|  | 7 | exp health services administration/ |
|  | 8 | (nurse or nurses or hospitals or nursing).ab,ti. |
|  | 9 | exp hospital units/ |
|  | 10 | or/8-9 |
|  | 11 | and/6-7,10 |
| Precise | 1 | exp "Outcome and Process Assessment (Health Care)"/ |
|  | 2 | exp Hospital Units/ |
|  | 3 | hospitals.ab,ti. |
|  | 4 | or/1-3 |
|  | 5 | (nurse or nurses).ab,ti. |
|  | 6 | staffing.ab,ti. |
|  | 7 | Nursing Staff, Hospital/ |
|  | 8 | or/6-7 |
|  | 9 | outcomes.ab,ti. |
|  | 10 | and/4-5,8-9 |
| Balanced | 1 | exp "Outcome and Process Assessment (Health Care)"/ |
|  | 2 | exp Hospital Units/ |
|  | 3 | hospitals.ab,ti. |
|  | 4 | or/1-3 |
|  | 5 | ((nurse or nurses) and staffing).ab,ti. |
|  | 6 | Nursing Staff, Hospital/ |
|  | 7 | or/5-6 |
|  | 8 | and/4,7 |

Table S3: Search strategies adjusted for In Process-Citations and Non-Medline records (untested)

| Sensitive | 1 | staff[tiab] OR staffing[tiab] OR organizational[tiab] OR skill mix[tiab] OR length of stay[tiab] OR medicare[tiab] |
| --- | --- | --- |
|  | 2 | nurse[tiab] OR nurses[tiab] OR hospitals[tiab] OR nursing[tiab] |
|  | 3 | medline[sb] |
|  | 4 | #1 AND #2 NOT #3 |
| Precise | 1 | nurse[tiab] or nurses[tiab] |
|  | 2 | staffing[tiab] |
|  | 3 | outcomes[tiab] |
|  | 4 | medline[sb] |
|  | 5 | (#1 AND #2 AND #3) NOT #4 |
| Balanced | 1 | hospitals[tiab] |
|  | 2 | (nurse[tiab] OR nurses[tiab]) AND staffing[tiab] |
|  | 3 | medline[sb] |
|  | 4 | #1 AND #2 NOT #3 |
